# The Long Noncoding RNA LINC00665 Facilitates c-Myc Transcriptional Activity *via* the miR-195-5p MYCBP Axis to Promote Progression of Lung Adenocarcinoma

**DOI:** 10.3389/fonc.2021.666551

**Published:** 2021-07-01

**Authors:** Anpeng Wang, Te Zhang, Wei Wei, Hui Wang, Zeyu Zhang, Wenming Yang, Wenjie Xia, Qixing Mao, Lin Xu, Feng Jiang, Gaochao Dong

**Affiliations:** ^1^ Department of Thoracic Surgery, Jiangsu Cancer Hospital & Jiangsu Institute of Cancer Research & The Affiliated Cancer Hospital of Nanjing Medical University, Nanjing, China; ^2^ Department of Geriatric Oncology, The First Affiliated Hospital of Nanjing Medical University, Nanjing, China; ^3^ Jiangsu Key Laboratory of Molecular and Translational Cancer Research, Cancer Institute of Jiangsu Province, Nanjing, China; ^4^ The Fourth Clinical College of Nanjing Medical University, Nanjing, China

**Keywords:** lncRNA, MiR-195-5p, MYCBP, LUAD, LINC00665

## Abstract

Long noncoding RNAs (lncRNAs) have recently received growing substantial attention in cancer research due to their important roles in various cancer types. However, the underlying mechanisms and functions of lncRNAs, especially in lung adenocarcinoma (LUAD), remain elusive. Based on pan-cancer screening analyses, we identified that the noncoding RNA LINC00665 was up-regulated in lung adenocarcinoma, which was subsequently confirmed in clinical samples and cell lines. Higher expression of LINC00665 was positively associated with poor prognosis and advanced T stage. Next, using gain- and loss- of function approaches, we revealed that LINC00665 promotes cell proliferation, cell migration, invasion, and suppresses cell apoptosis in LUAD through *in vitro* and *in vivo* experiments. Additionally, our findings showed that LINC00665 was predominately localized in the cytoplasm so as to interact with Ago2 protein, which could function as miRNA sponges. The results of bioinformatics prediction and RNA pull-down assay indicated that LINC00665 directly interacted with miR-195-5p. This was also confirmed by fluorescence colocalization. Furthermore, luciferase reporter assay demonstrated that Myc binding protein (MYCBP, also called AMY-1), which enhanced c-Myc transcriptional activity, was the target gene of LINC00665 dependent on miR-195-5p. Finally, rescue functional assay results uncovered that the oncogenic capability of LINC00665 was dependent on miR-195-5p and c-Myc transcriptional activity. In summary, this work elucidates that LINC00665 accelerates LUAD progression *via* the miR-195-5p/MYCBP axis by acting as a competing endogenous RNA (ceRNA), suggesting that LINC00665 may represent a potential therapeutic target for clinical intervention of LUAD.

## Introduction

Lung cancer is the leading cause of cancer-related deaths worldwide, with a large variation of the incidence and mortality across regions (1, 2). In particular, non-small cell lung cancer (NSCLC) accounts for around 85% of lung cancer cases (3). Notably, lung adenocarcinoma (LUAD) is the most prevalent pathohistological type of NSCLC (1–3). Despite the application of multimodal treatments, the overall survival of LUAD patients remains poor. Albeit great progression of diagnosis and treatment has recently been made. The related molecular biological mechanisms of LUAD are highly complex, numerous, and sophisticated (4, 5). Thus, the detailed mechanisms of LUAD malignant progression are largely unknown, requiring further clarification.

Long noncoding RNAs (lncRNAs) are a class of noncoding RNA transcripts that are more than 200 nucleotides in length, with no apparent protein-coding capacity (6, 7). It is well known that the abnormal expression of lncRNAs in LUAD is widespread, which play critical roles in tumor initiation and progression (8, 9). As an annotated human lncRNA, LINC00665 has been reported to occur primarily in the cytoplasm. Previous studies have established that LINC00665 acts as an oncogenic molecule in multiple human cancers, such as breast cancer (10), gastric cancer (11), hepatocellular carcinoma (12), prostate cancer (13), osteosarcoma (14), colorectal cancer (15), myeloma (16), glioma (17), ovarian cancer (18), and NSCLC (19). More importantly, LINC00665 can function as an oncogene by sponging different miRNAs, resulting in cancer-associated gene dysregulation in many cancers (14, 16, 19). For instance, it has been shown that the proliferation and migration ability of melanoma cells might be facilitated significantly by LINC00665 *via* modulating the miR224-5p/VMA21 axis (20). In lung cancer, it was found to promote LUAD angiogenesis by interacting with YB1 (21), while in NSCLC, cell proliferation and invasion were accelerated by regulating the miR-138-5p/E2F3 axis (19). Furthermore, acquired resistance to gefitinib in NSCLC might be caused by LINC00665 through recruiting EZH2, thereby activating the PI3K/AKT signaling pathway (22).

In this study, we first identified aberrant high expression lncRNA, LINC00665, in LUAD using tissue microarray technique. Results of qRT-PCR analysis confirmed that LINC00665 was upregulated in LUAD tissues by detecting the mRNA expression levels in LUAD tissues and adjacent normal lung tissue. We further observed that proliferation, invasion, and migration abilities of LUAD cells transfected with siRNA of LINC00665 were markedly decreased. Based on the mechanism involved, we identified that LINC00665 could act as a ceRNA to sponge miR-195-5p and indirectly increase the expression of MYCBP, an 11-kDa protein associated with transcription activation of MYC, in LUAD. Herein, downstream genes of the MYC signal pathway were also upregulated in LINC00665 overexpression. In brief, this study unveils an aberrantly activated axis, consisting of LINC00665/miR-195-5p/MYCBP, promoting LUAD malignant progression that might be a valuable and novel therapeutic target for the treatment of LUAD.

## Materials and Methods

### Clinical Samples

For this study, tumor tissues and paired normal lung tissues from LUAD patients who were undergoing thoracic surgery at Jiangsu Cancer Hospital (Nanjing, China) were collected and analyzed using qRT-PCR analysis. Notably, all tumors and matched normal tissues were confirmed by histopathological examination. This work was approved by the Ethics Committee of Jiangsu Cancer Hospital and met the ethical standards of the institution. All participants enrolled in this study provided written informed consent.

### Cell Culture and Treatment

Human LUAD cell lines, including HBE, A549, H1299, H1975, PC9, and SPCA-1 were purchased and maintained in RPMI 1640 (KeyGen) medium, while SPCA-1 and PC9 were cultured in DMEM (KeyGen) medium. Both media were supplemented with 10% FBS (Corning Technology) and incubated at 37°C in a humidified atmosphere containing 5% CO_2_.

### RNA Extraction and qRT-PCR

Briefly, the total RNA of tissue samples and cell lines was extracted using TRIzol (Invitrogen, USA) reagent following the manufacturer’s instructions. Then, about 1 μg of total RNA was reverse transcripted into cDNA using Invitrogen III transcriptase. After this, we performed real-time quantitative fluorescence PCR (qRT-PCR) using SYBR Green Master Mix (Applied Biosystems). GAPDH and U6 were used as internal control, while the ΔΔCT method was used for calculations. All data were evaluated using the StepOnePlus real-time PCR system (Applied Biosystems, USA). Primer sequences and other information are summarized in **Table S1**.

### siRNA Interference and Overexpression Plasmid Construction Assay

Specific siRNA sequence with a certain interference effect (si-LINC00665) was purchased from GenePharma (Shanghai, China), whereby hybrid siRNA was used as a negative control. On the other hand, human LINC00665, CMYC, and MYCBP overexpression plasmids were obtained from Genomeditech (Shanghai, China), where an empty vector (pcDNA3.1) was used as a negative control. Using Lipofectamine 3000 (Invitrogen, USA), LINC00665 overexpressed plasmid and siRNA were transfected into A549 and PC9 cells following the manufacturer’s protocol. Finally, total RNA and total protein were extracted 48 h after transfection and detected using qRT-PCR and Western blot analyses, respectively. All identified nucleotide sequences are depicted in **Table S1**.

### Real-Time Cell Analysis (RTCA)

Cell growth was monitored in real-time using the RTCA system. Briefly, RMI-1640 or DMEM medium, containing 10% FBS was placed in the lower chamber. Thereafter, cells were seeded in E-plates under conditions of 37°C and 5% CO2. Readings were taken every 15 minutes.

### EdU Assay

In brief, 50 mM EdU diluent medium was added to the cell samples for 2 h, while labeled proliferating cells were incubated with Apollo stain. DAPI was used to reverse stain the nucleus. Noticeably, a green signal of proliferating cells was observed under an inverted microscope (Leica DM4000 BLED).

### Flow Cytometric Analysis (FACS)

We performed flow cytometric analysis to assess the effect of LINC00665 downregulation and upregulation on cell cycle distribution and apoptosis. Specifically, A549 and PC9 cells were deposited in a 6-well plate. After incubation at 37°C overnight, cells were transfected with si-LINC00665 or control siRNA, plasmid LINC00665, and pcDNA3.1. Following reincubation for 24 h, the cells were collected and stained with the AnnexinV-FITC Apoptosis Detection Kit (BD Bioscience, Oxford, UK) as per the manufacturer’s guidelines. Next, we examined the cell cycle distribution and apoptosis rate using flow cytometry.

### Animal Experiment

Ten male BALB/c nude mice weighing 18-22 g were randomly divided into two groups for *in vivo* growth and metastasis tests. A549 cells (4×10^6^) were prepared by suspension with normal saline, and then subcutaneously injected into nude mice (5 mice in each group) or inoculated *via* tail vein with Sh-NC after transfection or Sh-LINC00665. Tumor volume was measured every 2 days after implantation. After 8 weeks, the mice were sacrificed and lung metastatic nodules were counted, where H&E staining confirmed the nodules as metastatic tumors. The protocols used in these studies were approved by the Animal Care and use Committee of the Cancer Hospital Affiliated to Nanjing Medical University. The animal tests were carried out in accordance with the regulations of China’s State Food and Drug Administration on animal care. The classification of animals is based on treatment only and does not exclude or include predetermined animals.

### RNA-Binding Protein Immunoprecipitation (RIP)

Cells were dissolved in ice-soluble buffer with RNase inhibitors. After each sample was centrifuged, the supernatant was preabsorbed with protein A/G incubation beads to eliminate nonspecific RNA binding for 1 h. After centrifugation, the A/G bead-end samples were then incubated with Argonaute-2 antibodies at 4°C overnight, and then 15 mg/ml of bead pre-blocked BSA was added to the antibody-lysate mixture and incubated for 2 h. The RNA/antibody complex was washed with RIPA buffer four times supplemented with a ribonuclease inhibitor protease inhibitor cocktail. Ultimately, RNA was extracted using TRIzol (Invitrogen) and analyzed using qRT-PCR according to the manufacturer’s protocol.

### Pull-Down Assay

Here, biotinylated LINC00665 probe (5’ – GAUACUGCUCCUUUUGCUGCUU - 3’) and miR195-5p probe (5’ – UAGCAGCACAGAAAUAUUGGC - 3’) were incubated with streptavidin magnetic beads (Life Technologies, USA) under conditions of 4°C for 2 h. After this, cell lysates were incubated with probe coated magnetic beads for 4 h at 4°C. Eventually, pull-down RNAs were extracted to detect RNA levels using qRT-PCR.

### Fluorescence *In Situ* Hybridization (FISH)

When the degree of cell fusion reached 40%, A549 and PC9 cells were immobilized with paraformaldehyde and then pre-hybridized and hybridized in a hybridization buffer. 5’Cy3 labeled LINC00665 targeted probe (5’ - AACACCGC+TAAGTCAC+TGACATTCC – 3’) and 3’FAM markers of the miR-195-5p targeted probe (5 - GCCAAUAUUUCUGUGCUGCUA - 3) by GenePharma (Shanghai, China) design synthesis. To detect the probe signal, a fluorescence *in situ* hybridization kit (GenePharma, China) was employed. Nuclei were stained with DAPI, while images were taken using a TCS SP5II confocal microscope (Leica Microsystems, Mannheim, Germany).

### Dual-Luciferase Reporter Assay

For luciferase reporter gene assay, a MYCBP 3’UTR clone in the plasmid was synthesized commercially using RiboBio to verify the activity of miR-195-5p. We purchased MiR-195-5p mimic from GenePharma, whereas the GV272 luciferase reporter gene construct containing wild-type (WT) or mutated MYCBP 3’UTR sequence was obtained from GeneChhem (Shanghai, China), where plasmid GV045 was used as an internal reference. The above constructs and renin luciferase plasmid were transfected into NSCLC cells as an internal control. After 24 h transfection of MYCBP 3’UTR clone plasmid, miR-195-5p mimics were reintroduced, followed by culturing of cells for 12 h, and then luciferase activity was detected. The luciferase values provided in the figure represent at least three independent transfection experiments performed independently.

### Western Blot Analysis

Briefly, total cell lysates were extracted from A549 and PC9 cells. To prepare cell lysates, phosphatase inhibitor and protease inhibitor cocktail (Thermo Fisher, USA) were supplemented with RIPA buffer (Thermo Fisher, USA). Afterward, protein concentration was determined using the Bicinchoninic Acid (BCA) protein detection kit (Thermo Fisher, USA). We separated protein samples (30μg) using 10–12% SDS-PAGE gels and then transferred them onto PVDF membranes. Following this, membranes were blocked with 5% skimmed milk and incubated overnight at 4°C with primary antibodies. Anti‐β‐actin, anti-c-Myc, and anti-MYCBP were acquired from Cell Signaling Technology (CST).

### Statistical Analysis

All statistical analyses were performed using GraphPad Prism, version 8.1 software. Qualitative variables were tested using the chi-square test or Fisher’s exact test. For continuous variables previously subjected to normal distribution tests, differences were compared using the Student t-test. Otherwise, variables are compared using nonparametric tests, and there is an outlier distribution. In addition, differences between groups were compared with analysis of variance (ANOVA) where applicable or nonparametric tests. Pearson correlation coefficient test was used for correlation analysis. Results are expressed as mean ± standard deviation (SD) unless stated otherwise. All statistical tests were 2-sided, where a value of P < 0.05 was considered statistically significant.

## Results

### LINC00665 Was Overexpressed in LUAD and Localized in the Cell Cytoplasm

**Figure 1:**
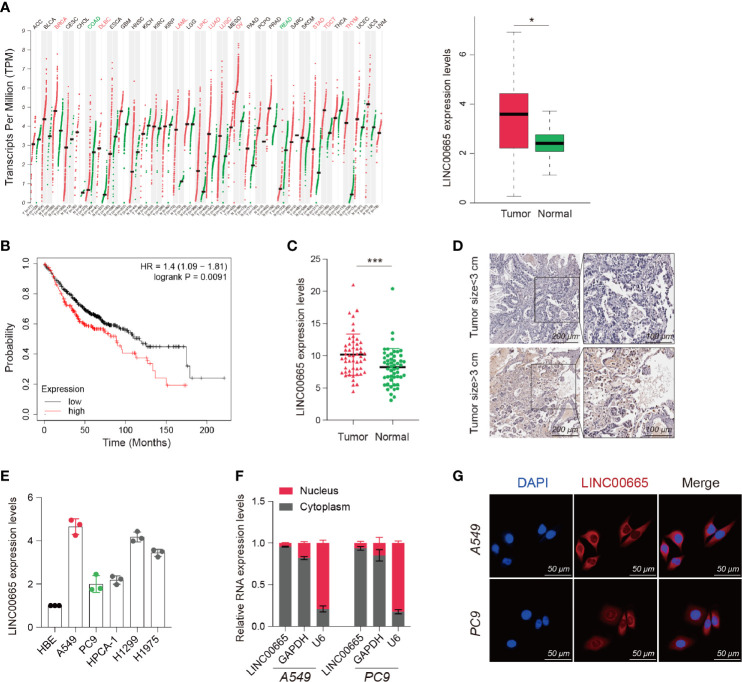
Identification, characterization, and clinical significance of LINC00665 in LUAD. **(A)** LINC00665 expression in multiple TUMOR and NORMAL tissues (Data from TCGA). **(B)** Kaplan-Meier survival analysis of LUAD patients with higher or lower levels of LINC00665. **(C)** LINC00665 expression in 52 pairs of LUAD and adjacent normal tissues measured using qRT-PCR. **(D)** LINC00665 FISH of 52 pairs of LUAD and adjacent normal tissues, tumor size ≥3cm or <3cm. **(E)** LINC00665 expression in HBE and multiple LUAD cell lines measured using qRT-PCR. **(F)** LINC00665 abundant in the cytoplasm of A549 and PC9 cells. GAPDH and U6 were used as positive controls in the cytoplasm and nucleus, respectively. **(G)** FISH assays showing the location of LINC00665 in A549 and PC9 cells, scale bars, 50 μm. *p < 0.05; ***p < 0.001.

Based on the Cancer Genome Atlas (TCGA) data analysis, we discovered that LINC00665 was overexpressed in multifarious tumors compared with normal tissues. Notably, LINC00665 was highly overexpressed in LUAD (**Figure 1A**). We also found that high expression of LINC00665 correlated with poor prognosis in LUAD (**Figure 1B**). Therefore, we selected LINC00665 for further study, where we infra measured its expression in 52 pairs of LUAD and para tumor tissues using qRT-PCR analysis. We subsequently uncovered that the expression of LINC00665 was considerably higher in LUAD tissues, particularly those with larger tumors (**Figures 1C, D**). We further unearthed that LINC00665 expression levels were markedly higher in the entire five LUAD cell lines than in HBE based on the qRT-PCR results (**Figure 1E**). We next investigated the LINC00665 subcellular location using FISH and qRT-PCR, where A549 and PC9 cells were selected for experiments. The results revealed that LINC00665 was predominately located in the cytoplasm (**Figures 1F, G**).

### LINC00665 Promotes Tumor Progression *In Vitro*

To examine the biological function of LINC00665 in LUAD, we first established LINC00665 overexpressed and LINC00665 knockdown LUAD cell lines. We herein employed a positive control vector (abovementioned) or small interfering RNAs (siRNAs) targeting LINC0066 to transfect A549 and PC9 cell lines, respectively. Additionally, we selected si-LINC00665#3 for subsequent experiments due to higher inhibition efficiency (**Figures S1A, B**).

**Figure 2:**
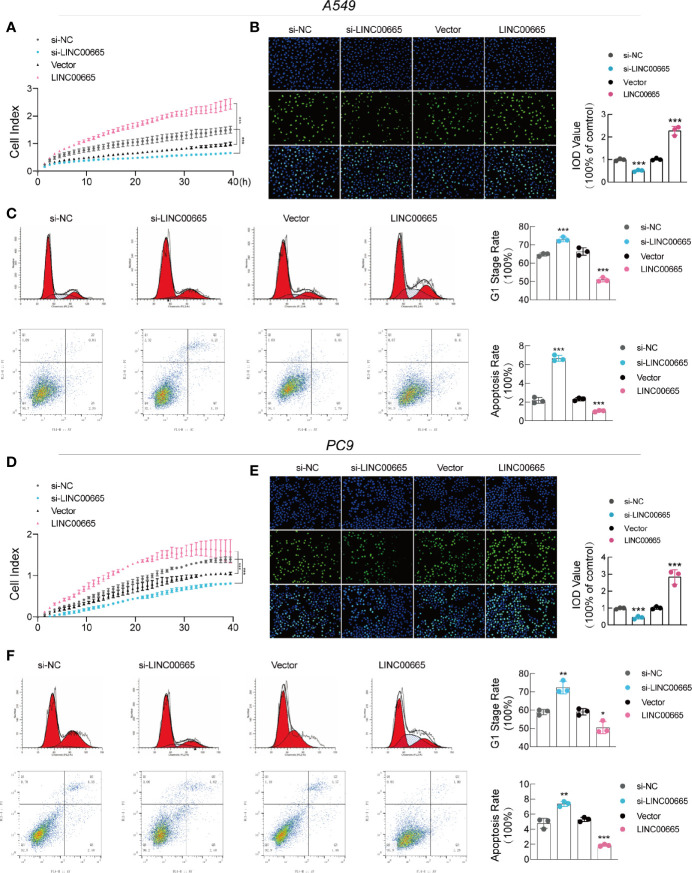
LINC00665 promotes LUAD cell growth and induces cell cycle arrest and apoptosis *in vitro.* **(A, B, D, E)** RTCA and EdU assays of A549 and PC9 cells with LINC00665 knockdown or overexpression. **(C, F)** Cell cycle and cell apoptosis rate of A549 and PC9 cells that received the indicated treatments analyzed with FACS. *p < 0.05; **p < 0.01; ***p < 0.001.

To determine whether LINC00665 was related to tumorigenesis, we performed RTCA and EdU assays. Interestingly, all our results confirmed that overexpressed LINC00665 significantly promoted the growth of LUAD cells, while knocking down LINC00665 suppressed the proliferation of LUAD cells (**Figures 2A, B, D, E**). This was also verified by the fluorescence-activated cell sorting (FACS) assay analysis that the overexpression of LINC00665 reduces G0/G1 cell arrest and apoptosis, while the reduction in its expression resulted in adverse results (**Figures 2C, F**). As shown in **Figure S2A**, the relative number of invading cells was markedly increased in the overexpression-LINC00665 group when compared with the empty vector group. Taken together, these findings demonstrate that LINC00665 promoted the proliferation and invasion of LUAD.

### LINC00665 Promotes Tumor Progression *In Vivo*

**Figure 3:**
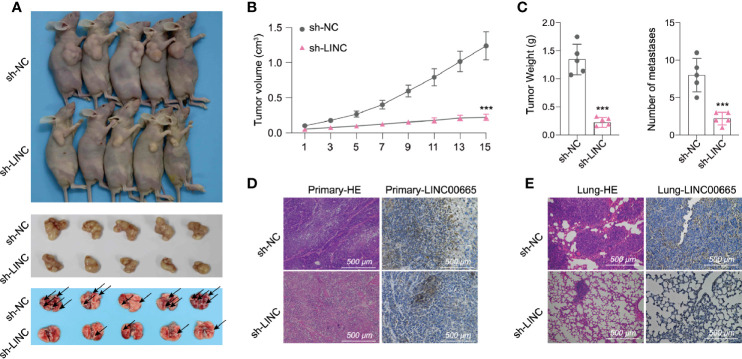
LINC00665 promotes tumor progression *in vivo.* **(A–C)** A549 cells transfected with sh-LINC00665 or sh-NC were subcutaneously injected into the armpit of a nude mouse (1×10^7^ cells per mouse, n = 5 each group). The volume, weight of subcutaneous xenograft tumors, and the number of lung metastasis of LUAD cells isolated from nude mice. **(D, E)** HE and LINC00665 FISH of xenograft or lung metastasis tumor after transfected with si-LINC00665 or sh-NC. ***p < 0.001.

To further explore whether LINC00665 participated in tumorigenesis *in vivo*, we designed short hairpin RNAs (shRNAs) that could specifically target LINC00665. We then subcutaneously injected A549 cells transfected with sh-NC or sh-LINC00665 into nude mice. Consequently, we identified that sh-LINC00665 significantly inhibited tumor growth and lung metastasis in mice (**Figures 3A–C**). We next assembled the subcutaneous tumor for further experiments. The HE and ISH (LINC00665) results established that the expression of LINC00665 in the sh-LINC00665 group was substantially lower in primary and lung metastasis tumors compared with that in the control group. Similarly, the lung metastasis of tumor cells was also significantly reduced (**Figures 3D, E**). Collectively, these results imply that LINC00665 may promote the growth and metastasis of LUAD *in vivo*.

### LINC00665 Acts as a Sponge of miR-195-5p

**Figure 4:**
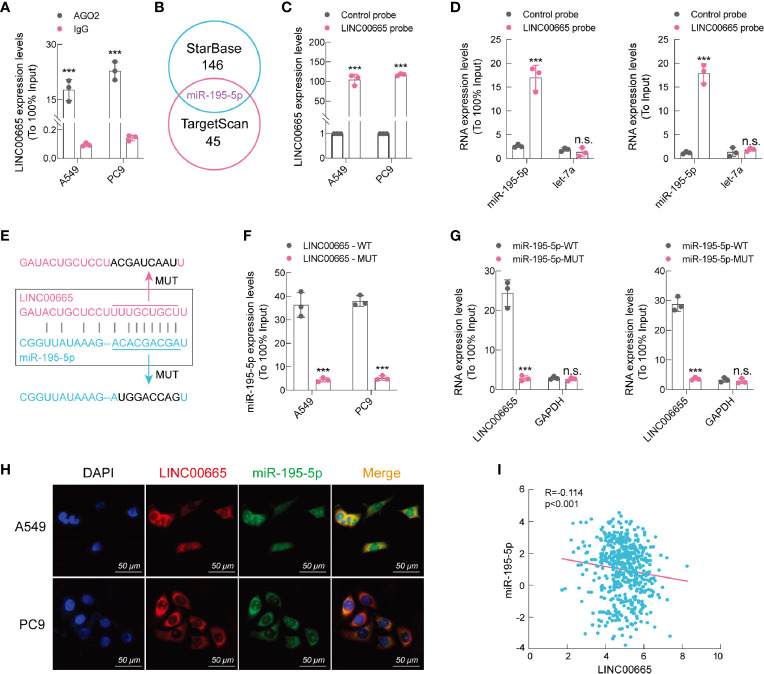
LINC00665 is a sponge of miR-195-5p. **(A)** RIP assay performed using AGO2 antibody, IgG served as a negative control. **(B)** Venn diagram showing the intersection of miRNA lists. **(C)** qRT-PCR results indicating that LINC00665 could be specifically enriched by the LINC00665 probe. **(D)** The relative expression levels of miR-195-5p and let-7 (negative control) detected using qRT-PCR in A549 and PC9 cell lysates. **(E)** Schematic graph illustrating the mutation of potential binding sites between LINC00665 and miR-195-5p. **(F)** miR-195-5p enriched by biotinylated wild-type or mutant LINC00665, and qRT-PCR used to determine the relative miR-195-5p levels. **(G)** LINC00665 enriched by biotinylated wild-type or mutant miR-195-5p, and qRT-PCR used to determine the relative miR-195-5p levels. **(H)** RNA FISH images showing the localization of LINC00665 and miR-195-5p in A549 and PC9 cells, scale bar, 50 μm. **(I)** Expression of LINC00665 and miR-195-5p that were negatively correlated (Data from TCGA). ***p < 0.001. n.s., no significance.

Numerous reports have shown that LncRNAs, among its various biological functions, exert “sponge-like” effects on diverse miRNAs (23–25). As mentioned earlier, we confirmed that LINC00665 was prevalently located in the cytoplasm. To explore the ability of LINC00665 to bind to miRNAs, we used A549 and PC9 cells to perform the RIP experiment. Our findings demonstrated that LINC00665 was markedly enriched under the action of anti-AGO2 antibody (**Figure 4A**). Afterward, we screened miR-195-5p through bioinformatic analysis databases (**Figure 4B**). The direct interaction between miR-195-5p and LINC00665 was assessed using RNA pull-down assays with a specific biotin-labeled anti-LINC00665 probe. As anticipated, we found that the biotin-probe can specifically bind to LINC00665 (**Figure 4C**), while the expression of miR-195-5p co-precipitated with the biotin-labeled anti-LINC00665 probe was remarkably increased compared to that with the control probe in both A549 and PC9 cells (**Figure 4D**). We further conducted a pull-down experiment of mutant biotinylated LINC00665 with miR-195-5p (**Figure 4E**), which revealed that the enrichment of miR-195-5p in the capture portion of mutant LINC00665 was significantly decreased than that of the wild-type LINC00665, and so was the mutant miR-195-5p probe (**Figures 4F, G**). Moreover, we performed FISH assay for LINC00665 and miR-195-5p subcellular location. The results uncovered that miR-195-5p co-localized with LINC00665 in the cytoplasm (**Figure 4H**). We also found a strong negative correlation (R^2^ = -0.114, p < 0.001, data from TCGA) between LINC00665 and miR-195-5p in LUAD tissues (**Figure 4I**). In summary, these outcomes suggest that LINC00665 can directly bind miR-195-5p.

### LINC00665 Sponged miR-195-5p to Regulate MYCBP

**Figure 5:**
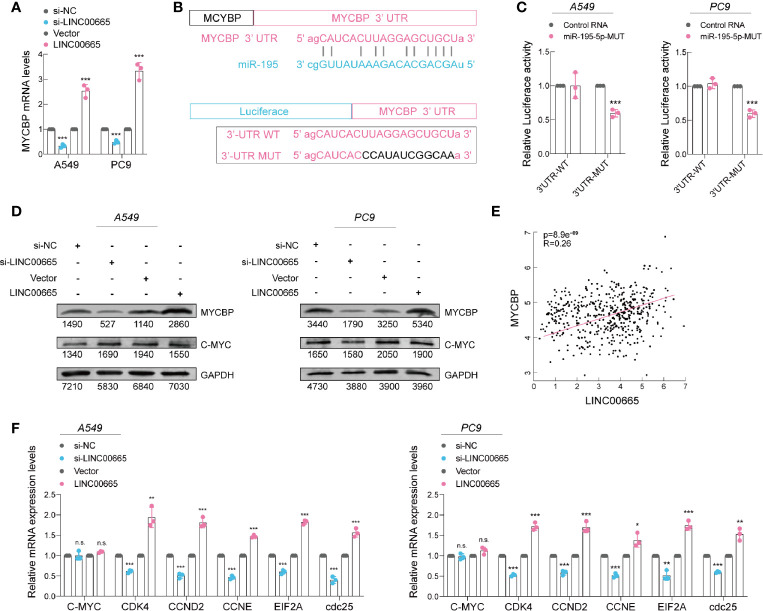
miR-195-5p promotes progression of LUAD *via* targeting MYCBP. **(A)** MYCBP mRNA expression level in 549 and PC9 cells after knockdown or overexpression of LINC00665 determined using qRT-PCR. **(B)** Upper panel: miR-195-5p can bind to the 3’-UTR of MYCBP mRNA; Lower panel: Schematic graph illustrating the mutation of potential binding sites between miR-195-5p and the 3′-UTR regions of MYCBP. **(C)** MYCBP 3’-UTR and miR-195-5p binding site analyzed with dual-luciferase reporter assay. **(D)** MYCBP and C-Myc protein expression levels detected using western blot analysis in A549 and PC9 cells with knockdown or overexpression of LINC00665. **(E)** Expression of LINC00665 and MYCBP that are positively correlated (Data from TCGA). **(F)** C-Myc downstream mRNA levels measured using qRT-PCR in A549 and PC9 cells after LINC00665 knockdown or overexpression. *p < 0.05; **p < 0.01; ***p < 0.001. n.s., no significance.

Recent studies have concluded that lncRNA can act as a ceRNA to absorb microRNA and then stimulate mRNA expression, which are the targets of miRNAs (26). Analysis using three publicly available algorithms, we discovered that MYCBP mRNA could be a target of miR-195-5p (**Figure S2B**). In particular, MYCBP is a novel c-Myc binding protein that stimulates the transcription activity of c-Myc (27–29), which is an important oncogene that can promote cell proliferation (30–32). We then employed qRT-PCR to detect the expression level of MYCBP mRNA in A549 and PC9 cell lines after LINC00665 silenced or overexpressed. Remarkably, the results demonstrated that the expression of MYCBP mRNA was significantly influenced by LINC00665 (**Figure 5A**). Furthermore, we conducted a dual-luciferase reporter assay, whereby the luciferase activity was significantly decreased when co-transfected with miR-195-5p and MYCBP wild-type reporter plasmids (**Figures 5B, C**). Taken together, based on these findings, we noted that MYCBP was absolutely a direct target of miR-195-5p.

Then, we further detected the MYCBP protein levels in A549 and PC9 cell lines after LINC00665 silenced or overexpressed using western blot assays. We found a lower level of MYCBP in the LINC00665-silenced group, whereas its higher level was observed in the LINC00665-overexpressed group in both A549 and PC9 cells. As expected, the c-Myc expression level exhibited no obvious change (**Figure 5D**). Furthermore, we discovered a notable positive correlation (R^2^ = 0.26; P<0.001; data from TCGA) between LINC00665 and MYCBP mRNA in LUAD tissues (**Figure 5E**). Overwhelming evidence demonstrates that MYCBP acts by modulating the transactivation activity of c-Myc to enhance the transcription of c-Myc downstream E-BOX containing genes (27, 33, 34). We also verified the corresponding results in these genes (**Figure 5F**).

In general, these findings imply that LINC00665 functions as a miR-195-5p sponge, regulating MYCBP expression and promoting tumor growth in LUAD.

### LINC00665 and c-Myc Reversed the Tumor-Suppressor Role of miR-195-5p in LUAD

**Figure 6:**
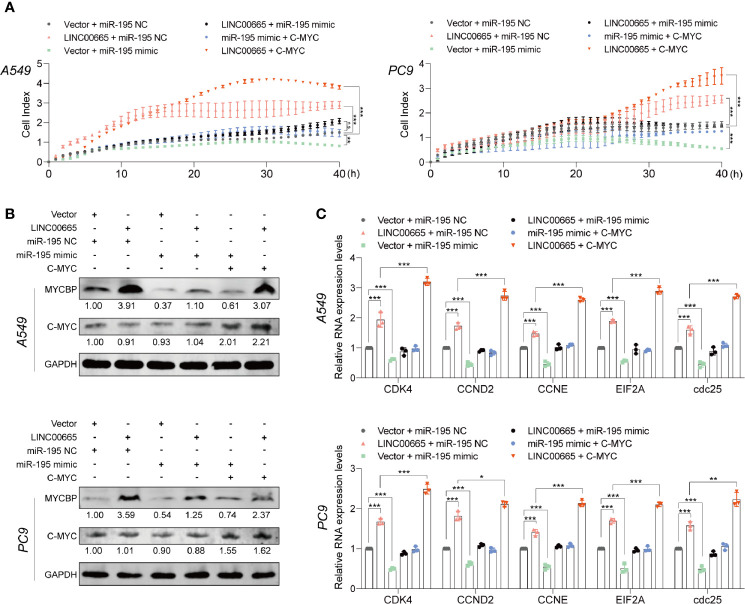
miR-195-5p reverses the tumor-promoted roles of LINC00665 or c-Myc in A549 and PC9 cells. **(A)** RTCA assay performed in A549 and PC9 cells after LINC00665 knockdown or overexpression and cotransfection with miR-195-5p inhibitor or mimics. **(B)** MYCBP and C-Myc protein levels measured in A549 and PC9 cells after LINC00665 knockdown or overexpression and cotransfection with miR-195-5p inhibitor or mimic or C-Myc. **(C)** C-Myc downstream mRNA levels measured with q-RT-PCR in A549 and PC9 cells after LINC00665 knockdown or overexpression and cotransfection with miR-195-5p inhibitor or mimic or C-Myc. *p < 0.05; **p < 0.01; ***p < 0.001. n.s., no significance.

Importantly, miR-195-5p has been reported to play an essential role in regulating NOTCH2-mediated tumor cell EMT, thereby affecting IL-4-related M2-like TAM polarization in CRC (35). Here, we observed that overexpression of LINC00665 significantly promoted cell proliferation, while the promotion could be reversed by co-transfection with miR-195-5p mimic. Meanwhile, we noted that the inhibition of cell proliferation by miR-195-5p can be reversed by co-transfection with c-Myc (**Figure 6A**). On the other hand, the expression level of MYCBP protein was substantially elevated in the LINC00665+miR-195-NC group, but this elevation could not be reversed by co-transfecting with miR-195-5p mimic as expected, thus c-Myc levels were not significantly changed (**Figure 6B**). Of note, we confirmed that c-Myc downstream E-BOX containing genes can be regulated by LINC00665, miR-195-5p, and c-Myc (**Figure 6C**).

## Discussion

Aberrantly activated dysregulation of lncRNAs and miRNAs has been elucidated to participate in pathogenesis, development, and metastasis in LUAD (9). In 2018, LINC00665 was initially identified as a lncRNA, which is highly overexpressed in hepatocellular carcinoma (HCC) and associated with poor progression in HCC patients (36). Recent studies have established that EZH2 can be recruited by LINC00665, resulting in PI3K/AKT pathway activation in NSCLC with acquired resistance to gefitinib (22). Similarly, the Wnt signaling pathway was also activated in gastric cancer malignant progression *via* LINC00665 (22). In LUAD, LINC00665 was reported to function as ceRNA *via* sponging miR-98 to activate the AKR1B10-ERK signaling pathway (37). Apart from this, CIP2A-BP, a micro peptide encoded by LINC00665, was found to inhibit triple-negative breast cancer progression elsewhere (38). In this present investigation, our results suggested that LINC00665 was up-regulated in LUAD tumor tissues compared with adjacent normal lung tissue. Further, we determined that LINC00665 is capable of enhancing LUAD cell proliferation, invasion, and migration based on *in vitro* and *in vivo* experiments, indicating a tumor promoter role in LUAD. However, the involved precise molecular biological mechanisms need to be further investigated in LUAD, and albeit LINC00665 has recently been reported in several studies.

Based on previous reports, proper subcellular localization of lncRNA is crucial for biological function (39). Mounting evidence posits that lncRNA located in the cytoplasm is involved in gene regulation at the post-transcriptional level *via* acting as ceRNAs and protecting the target mRNAs from repression (40). In this study, we unearthed that LINC00665 was preferentially localized in the cytoplasm using RNA FISH and cell cytoplasmic/nuclear fractionation assays in LUAD cells. indicating its potential for functioning as a miRNA sponge. Findings of bioinformatics analysis revealed that binding sites of miR-195-5p existed in the LINC00665 sequence, which was subsequently confirmed by RNA pull-down, RIP, and luciferase reporter assays. Moreover, correlation analysis results elucidated that LINC00665 negatively correlates with miR-195-5p in LUAD, which partially reversed LINC00665 overexpression-mediated malignant phenotypes, such as proliferation, invasion, and migration of cancer cells in LUAD. So far, this study suggests that LINC00665 could function as a ceRNA *via* sponging miR-195-5p in LUAD.

MYCBP, a c-MYC-binding protein, has been found to regulate the transcriptional activity of MYC and stimulate the activation of E box-dependent transcription through MYC. Elsewhere, MYCBP was observed to be upregulated in HCC, repressed by EYA4 to inhibit malignant progression (41). Further empirical studies have revealed that MYCBP interacts with SPAG5 enhancing c-MYC transcriptional activity in triple-negative breast cancer (42). Besides, a recent study reported that MYCBP was inhibited by miR-22, showing a therapeutic target potential in acute myeloid leukemia (43). However, the underlying physiological role or molecular function of MYCBP in LUAD remains enigmatic. More importantly, this study identified MYCBP as a target protein of the LINC00665/miR-195-5p axis based on the following findings. First, MYCBP was identified to be a target gene of miR-195-5p in LUAD cell lines through bioinformatic prediction. Second, the results of correlation analysis showed that LINC00665 was positively correlated with MYCBP in LUAD, which could be partially reversed by miR-195-5p overexpression, indicating a LINC00665/miR-195-5p/MYCBP axis in LUAD. Finally, key downstream oncogenes, namely, CDK4, CCND2, CCNE, EIF2A, and cdc25, involved in the c-MYC signaling pathway were up-regulated in LINC00665 overexpressed LUAD cell lines, which could also be partially inhibited by overexpression of miR-195-5p hence a key transcription factor of c-MYC.

**Figure 7:**
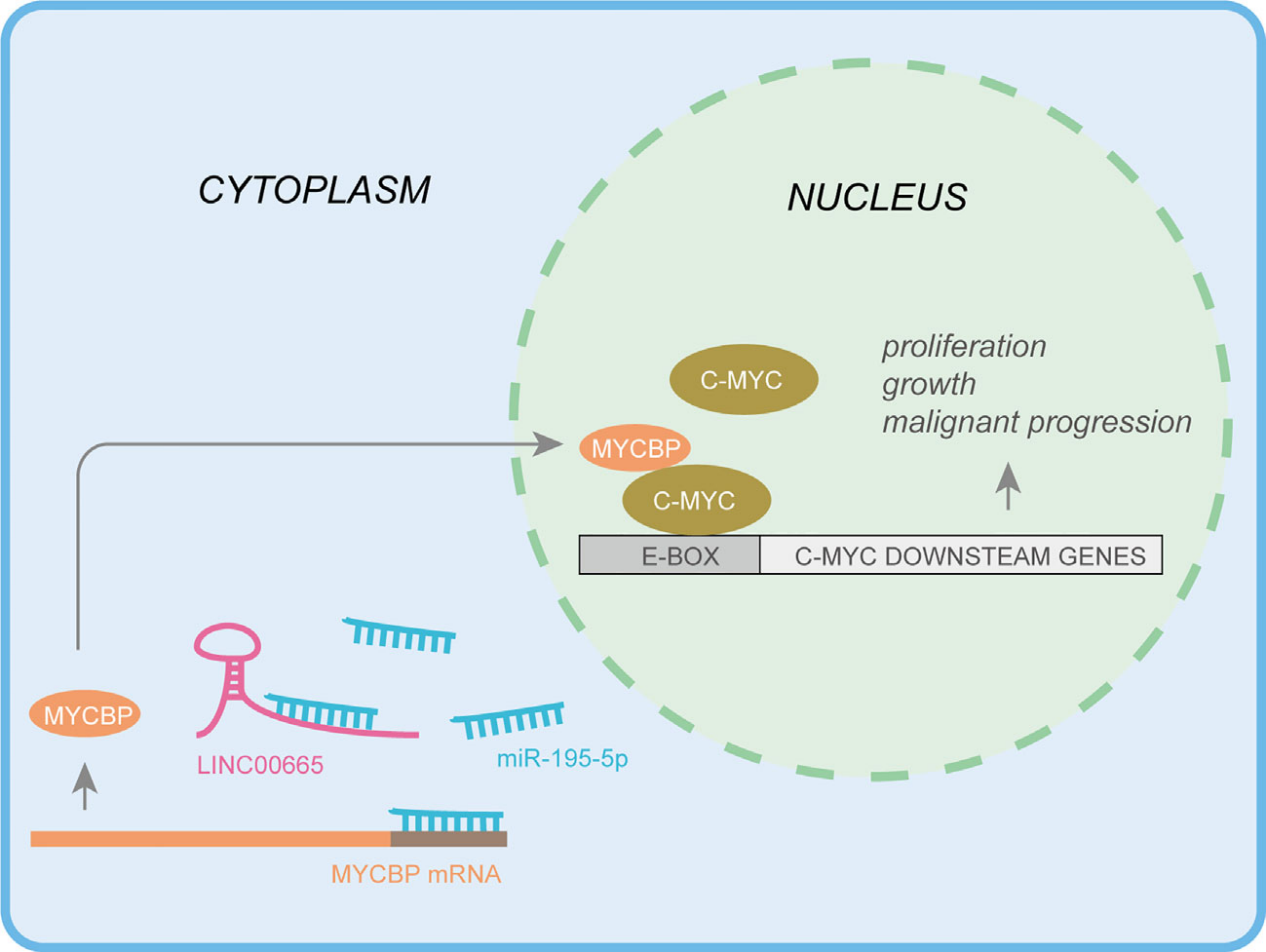
Integrated model depicting LINC00665 driving LUAD progression.

In conclusion, this work demonstrates that LINC00665 could function as a ceRNA by sponging miR-195-5p to up-regulate MYCBP, resulting in the activation of the canonical c-MYC pathway in LUAD (**Figure 7**). Therefore, the findings of this study provide novel evidence supporting LINC00665 as a valuable therapeutic target in LUAD. Of noteworthy, this study is the first to target MYCBP in LUAD *via* LINC00665/miR-195-5p/MYCBP axis.

## Data Availability Statement

The datasets presented in this study can be found in online repositories. The names of the repository/repositories and accession number(s) can be found below: https://www.ncbi.nlm.nih.gov/, GSE104854.

## Ethics Statement

The studies involving human participants were reviewed and approved by the Ethics Committee of Jiangsu Cancer Hospital. The patients/participants provided their written informed consent to participate in this study. The animal study was reviewed and approved by the Animal Care and use Committee of the Cancer Hospital Affiliated to Nanjing Medical University.

## Author Contributions

FJ, GD, and LX conceived and designed the experiments. AW and TZ designed and carried out most of the experiments, and manuscript writing WW advised on experimental design and participated in the analysis of data. HW, WY, WX, and QM helped in cell culture and sample collecting. All authors contributed to the article and approved the submitted version.

## Funding

This study was supported by the grants from the National Natural Science Foundation of China (Grant No. 82073211, 82002434, 82003106); The Project of Invigorating Health Care through Science, Technology and Education, Jiangsu Provincial Medical Innovation Team (CXTDA2017002); The Project of Invigorating Health Care through Science, Technology and Education, Jiangsu Provincial Medical Outstanding Talent (JCRCA2016001); Young Talents Program of Jiangsu Cancer Hospital.

## Conflict of Interest

The authors declare that the research was conducted in the absence of any commercial or financial relationships that could be construed as a potential conflict of interest.
