# Supplementary figures and images for: The Long Noncoding RNA LINC00665 Facilitates c-Myc Transcriptional Activity via the miR-195-5p MYCBP Axis to Promote Progression of Lung Adenocarcinoma
Source: Front Oncol. 2021 Jul 1;11:666551. doi: 10.3389/fonc.2021.666551 (PMC8281894; doi:10.3389/fonc.2021.666551)

A

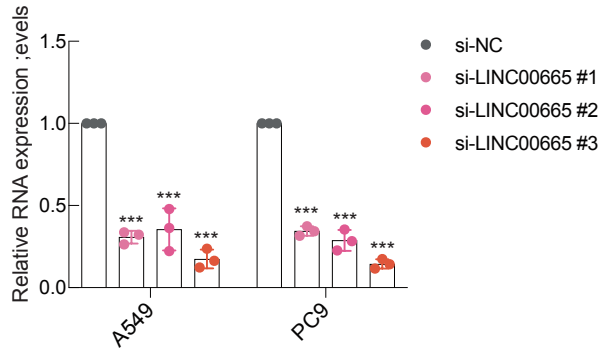

B

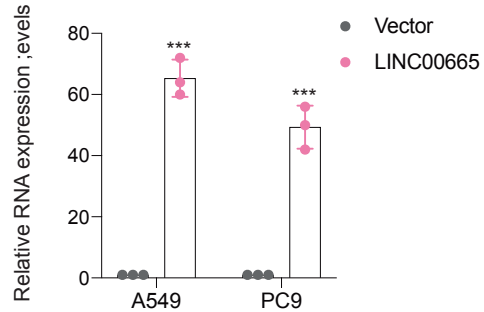

Supplement: Supplementary file 1 [file DataSheet_1.pdf]

**A****PC9**

Empty Vector

LINC00665

Transwell

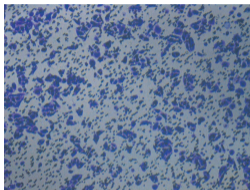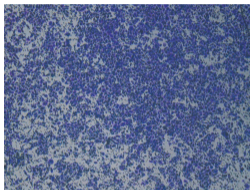

Matrigell

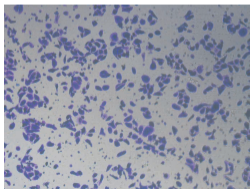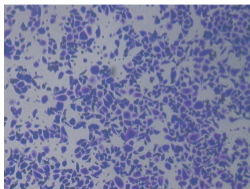**B**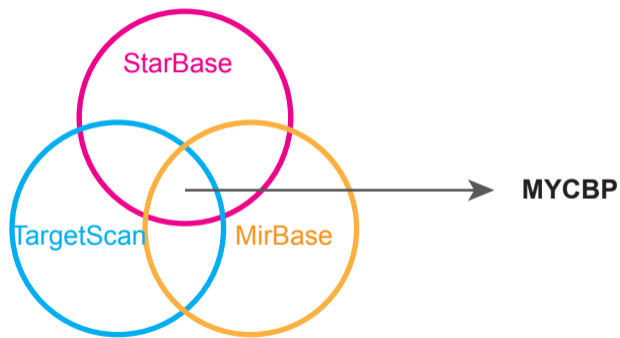

Supplement: Supplementary file 2 [file DataSheet_2.pdf]
