# Supplementary material for: The Long Noncoding RNA LINC00665 Facilitates c-Myc Transcriptional Activity via the miR-195-5p MYCBP Axis to Promote Progression of Lung Adenocarcinoma
Source: Front Oncol. 2021 Jul 1;11:666551. doi: 10.3389/fonc.2021.666551 (PMC8281894; doi:10.3389/fonc.2021.666551)
Supplement: Supplementary file 3 [file Table_1.docx]

| **Table S1: The sequences of primers, oligonucleotides and probes used in this study.** | | |
| --- | --- | --- |
| **Primers for PCR (5’-3’)** | | **concentration** |
| LINC00665-F | ACTCTGTGTCCTTCTCTGCC | 1μmol |
| LINC00665-R | AACCACCTCAGATTCTCCAAAA | 1μmol |
| GAPDH-F | GGAGCGAGATCCCTCCAAAAT | 1μmol |
| GAPDH-R | GGCTGTTGTCATACTTCTCATGG | 1μmol |
| U6-F | CTCGCTTCGGCAGCACA | 1μmol |
| U6-R | AACGCTTCACGAATTTGCGT | 1μmol |
| MYCBP-F | ATGGCCCATTACAAAGCCG | 1μmol |
| MYCBP-R | TTTCTGGAGTAGCAGCTCCTAA | 1μmol |
| CCND2-F | ACCTTCCGCAGTGCTCCTA | 1μmol |
| CCND2-R | CCCAGCCAAGAAACGGTCC | 1μmol |
| CDK4-F | ATGGCTACCTCTCGATATGAGC | 1μmol |
| CDK4-R | CATTGGGGACTCTCACACTCT | 1μmol |
| LDHA-F | ATGGCAACTCTAAAGGATCAGC | 1μmol |
| LDHA-R | CCAACCCCAACAACTGTAATCT | 1μmol |
| NCL-F | GGTGGTCGTTTCCCCAACAAA | 1μmol |
| NCL-R | GCCAGGTGTGGTAACTGCT | 1μmol |
| EIF2A-F | CCGCTCTTGACAGTCCGAG | 1μmol |
| EIF2A-R | GCAGTAGTCCCTTGTTAGTGACA | 1μmol |
| miR-195-5p-F | Ribobio | 0.2μmol |
| miR-195-5p-R | Ribobio | 0.2μmol |
| **SiRNAs (sense sequence)** | | **concentration** |
| si-LINC00665 | 5'-UCCUCAGUCUUGGGCUAUUTT-3' | 50nmol |
| si-NC | 5'-UUCUCCGAACGUGUCACGUTT-3 | 50nmol |
| **MiR-195-5p mimics and inhibitors (** **sense sequence)** | | **concentration** |
| mimics NC | UAGCAGCACAGAAAUAUUGGC | 50nmol |
| inhibitor NC | CAGUACUUUUGUGUAGUACAA | 50nmol |
| miR-195-5p mimic | UAGCAGCACAGAAAUAUUGGC | 50nmol |
| miR-195-5p inhibitor | AUCGUCGUGUCUUUAUAACCG | 50nmol |
| biotin-miR-195-5p wild-type | UAGCAGCACAGAAAUAUUGGC | 50nmol |
| biotin-miR-195-5p mutant | UGACCAGGUAGAAAUAUUGGC | 50nmol |
| LINC-00665-probe | GAUACUGCUCCUUUUGCUGCUU | 50nmol |
| LINC-00665-probe mutant | GAUACUGCUCCUACGAUCAAUU | 50nmol |
| **ShRNAs (Vector pGFP-u6)** | | **concentration** |
| sh-LINC00665 | CACCGGTGTGCATTCAAGGAAGCCAGCTCGAGCTCCTCAGTCTTGGGCTATTTTTTTTTGGATC | 2μg |
| **Overexpression RNAs (Vetor pcDNA 3.1)** | | **concentration** |
| LINC00665 | hanbio | 2μg |
| C-MYC | hanbio | 2μg |
